# Porcine circovirus 2 (PCV2) population study in experimentally infected pigs developing PCV2-systemic disease or a subclinical infection

**DOI:** 10.1038/s41598-020-74627-3

**Published:** 2020-10-20

**Authors:** Florencia Correa-Fiz, Giovanni Franzo, Anna Llorens, Eva Huerta, Marina Sibila, Tuija Kekarainen, Joaquim Segalés

**Affiliations:** 1 Centre de Recerca en Sanitat Animal (CReSA, IRTA-UAB), IRTA, Bellaterra, Spain; 2 OIE Collaborating Centre for the Research and Control of Emerging and Re-Emerging Swine Diseases in Europe (IRTA-CReSA), Bellaterra, Barcelona, Spain; 3 Department of Animal Medicine, Production and Health (MAPS), University of Padua, Legnaro, PD Italy; 4 Departament de Sanitat i Anatomia Animals, Facultat de Veterinària, UAB, Bellaterra, Spain; 5 Present Address: Kuopio Center for Gene and Cell Therapy, Microkatu 1, Kuopio, Finland

**Keywords:** DNA, Data processing, Experimental evolution

## Abstract

Porcine circovirus 2 (PCV2) is a single stranded DNA virus with one of the highest mutation rates among DNA viruses. This ability allows it to generate a cloud of mutants constantly providing new opportunities to adapt and evade the immune system. This pig pathogen is associated to many diseases, globally called porcine circovirus diseases (PCVD) and has been a threat to pig industry since its discovery in the early 90’s. Although 11 ORFs have been predicted from its genome, only two main proteins have been deeply characterized, i.e. Rep and Cap. The structural Cap protein possesses the majority of the epitopic determinants of this non-enveloped virus. The evolution of PCV2 is affected by both natural and vaccine-induced immune responses, which enhances the genetic variability, especially in the most immunogenic Cap region. Intra-host variability has been also demonstrated in infected animals where long-lasting infections can take place. However, the association between this intra-host variability and pathogenesis has never been studied for this virus. Here, the within-host PCV2 variability was monitored over time by next generation sequencing during an experimental infection, demonstrating the presence of large heterogeneity. Remarkably, the level of quasispecies diversity, affecting particularly the Cap coding region, was statistically different depending on viremia levels and clinical signs detected after infection. Moreover, we proved the existence of hyper mutant subjects harboring a remarkably higher number of genetic variants. Altogether, these results suggest an interaction between genetic diversity, host immune system and disease severity.

## Introduction

The viral life-cycle is essentially dominated by a complex balance of interaction and competition with the host. To elude host defenses, different viral families have developed several strategies. These strategies can be broadly categorized into two non-mutually exclusive classes: (i) the ability to down-regulate the host immune responses, and (ii) the ability to evade the mounted immunity. While large complex viruses possess a broad set of proteins to interact with the host immune pathways, relatively simple RNA and single strand DNA (ssDNA) viruses, rely mainly on the genesis of new variants able to escape the immunity of the host and thereafter, potentially, of a broader population. In fact, the estimated evolutionary rate of these viruses is in the range of 10^–3^–10^–6^ substitution/site/year, corresponding to an average of one mutation per replication cycle^1–5^. The continuous genesis of a plethora of new mutants provides a huge substrate in which natural selection can act, favoring those more adapted to the new environment. Porcine circovirus type 2 (PCV2) belongs to this viral group, displaying one of the higher evolutionary rate among DNA viruses (~ 10^–3^)^6,7^, in the range of RNA viruses. Initially described in systemically diseased animals in 1991 in Canada^8^, PCV2 infection has been thereafter reported worldwide in association to several clinical conditions, including PCV2-systemic disease (PCV2-SD), PCV2-reproductive disease and porcine dermatitis and nephropathy syndrome, which are globally overall called Porcine circovirus diseases (PCVD)^9^. PCV2 is featured by a circular ssDNA genome of about 1.7 kb coding for a limited protein number. Although at least 11 ORF have been putatively identified, only 6 of them have been characterized to code biologically relevant proteins^10^. Particularly, only ORF1 and ORF2 are essential for PCV2 replication. ORF1 encode, through alternative splicing, two proteins (Rep and Rep’) involved in the rolling circle replication process. ORF2 protein, on the other hand, is the only PCV2 structural protein constituting the icosahedral viral capsid. Therefore, it plays a pivotal biological role being involved in viral attachment but also containing the majority of epitopic determinants.

The other ORFs (ORFs 3 to 6) coding for non-structural proteins not essential for viral replication, are involved in regulating cell apoptosis and interacting with the transcription and function of other viral/host proteins, potentially enhancing viral replication, survival, and even immune evasion^11–14^. A significant action of the host immune response, both natural and vaccine-induced^7,15^, has been demonstrated to affect PCV2 evolution, likely contributing to enhance the high genetic variability of this worldwide distributed virus. A noteworthy intra-host variability has been demonstrated in PCV2 infected subjects^16^, which could be of great relevance by constituting a substratum for larger scale evolution (i.e. population level). Additionally, a role on PCVD pathogenesis could also be hypothesized. In fact, PCV2 is able to establish relatively long-lasting infections, persisting for weeks or months. Hence, some mechanisms of immune evasion can be expected, as suggested by the higher within-host heterogeneity in the more immunogenic Cap coding gene (ORF 2) compared to other genomic regions^16^. Additionally, PCV2 interaction with the host immune system is particularly complex, since both immune activation and suppression have been proven to enhance viral replication, correlating with the emergence of overt clinical signs^17,18^. While an association between intra-host variability and pathogenesis or disease progression has been demonstrated for several human and animal infections^19–22^, such issue has never been investigated for PCV2.

Therefore, in the present study, the within-host PCV2 variability was monitored over time by next generation sequencing (NGS) in experimentally infected animals showing different clinical outcomes and viral loads.

## Results

### Pathological and virological characteristics of studied animals

An experimental inoculation of 6-weeks-old healthy pigs with very low PCV2 antibody titers (≤ 1:80), was done with PCV2b as inoculum. Serum samples from 23 pigs were used for high throughput sequencing. These animals were selected based on two main variables: PCV2 viremia measured by real-time quantitative PCR^23^ and fulfillment or not of the diagnostic criteria for PCV2-SD^9^. Viremia was considered high when ≥ 7 log_10_ PCV2 genome copies/mL were detected in serum at 3 weeks post-inoculation (wpi), and low when this was < 7 log_10_ PCV2 genome copies/mL in serum. PCV2-SD was diagnosed when animals displayed weight loss and fulfilled the histopathological criteria of moderate to severe lymphocyte depletion (LD) and granulomatous inflammation (GI) of lymphoid tissues, and harbored moderate to high amount of PCV2 nucleic acid measured by in situ hybridization (ISH) in these tissues (mean pathological combined score > 1.5). Animals that did not accomplish the abovementioned definition were considered as non-PCV2-SD (mean pathological combined score < 1.5).

These 23 animals were divided in the following three groups: Group 1 (PCV2-SD, n = 8); Group 2 (low viremia, non-PCV2-SD, n = 8); and Group 3 (high viremia, non-PCV2-SD, n = 7). A summary of the main characteristics of the different groups of pigs selected for metagenomic analyses (n = 23) is indicated in Table 1.

**Table 1:** Main characteristics of selected pigs for genetic analyses.

Characteristics	Group 1 (n = 8)	Group 2 (n = 8)	Group 3 (n = 7)
**PCV2-SD diagnosis**	Yes	No	No
**PCV2 load (log genome copies/mL) at 3 wpi**	4.94–7.83	4.64–6.72	7.01–9.54
**Histopathological score range**	1.5–3	0.25–1.08	0.17–1.08
Average daily weight gain	230 ± 83 g/day	480 ± 136 g/day	470 ± 81 g/day

The characteristics that were used as selection criteria of inclusion are bolded.

### Viral and antibody titers

**Figure 1:**
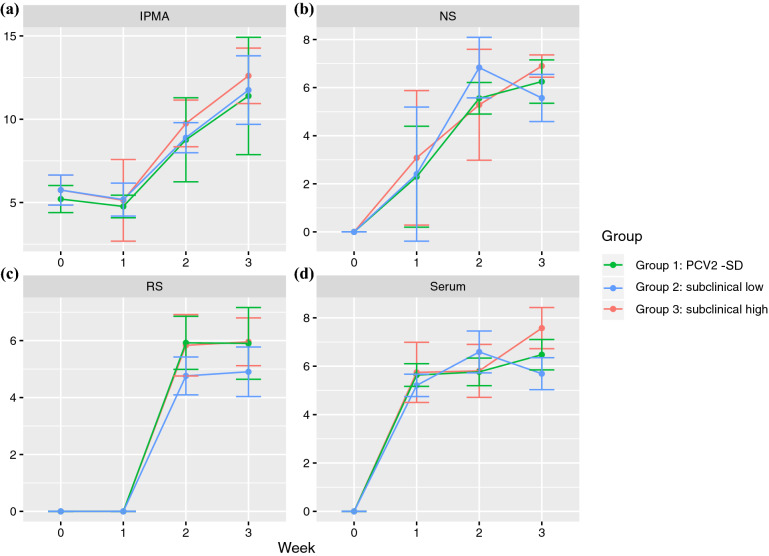
The average and 95% confidence intervals of log_2_ antibody titers (IPMA) measured in sera (**a**) and log_10_ PCV2 copies in 1 ml of PBS in nasal (**b**) and rectal swab (**c**) suspensions and 1 ml of serum (**d**) are reported for each wpi. Different groups have been color-coded.

Animals displayed detectable viral loads in serum, as well as in nasal and rectal swabs during the study period (Fig. 1). Particularly, PCV2 DNA could be detected from 1 wpi in serum and nasal swabs (NS), and from 2 wpi in rectal swabs (RS).

Seroconversion was evident from 2 wpi onwards in all studied animals and featured by a significant (*P* < 0.05) increase in antibody titers (Fig. 1a). A significant (*P* < 0.05) increase viral titer was observable in all groups at 1 wpi in serum and at 2 wpi in NS and RS. No significant differences were observed among groups except for PCV2 titer in serum between group 2 and 3, at 3 wpi.

### Viral subpopulation variation

**Figure 2:**
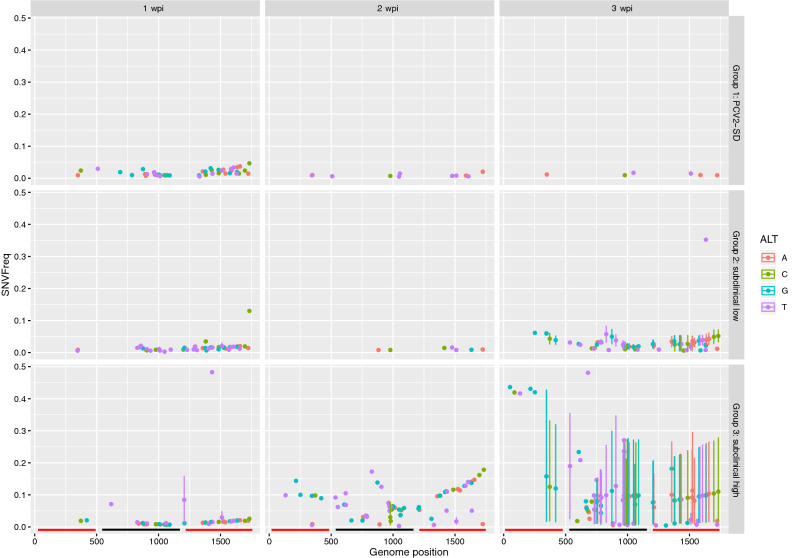
The SNV frequency is reported for each genome position. The regions corresponding to the ORF1 and ORF2 have been represented as red and black lines, respectively. Error bars indicate the presence of multiple subjects harboring the same SNV and describe the frequency variability. The wpi are reported as columns while the different groups are reported in separate rows.

**Figure 3:**
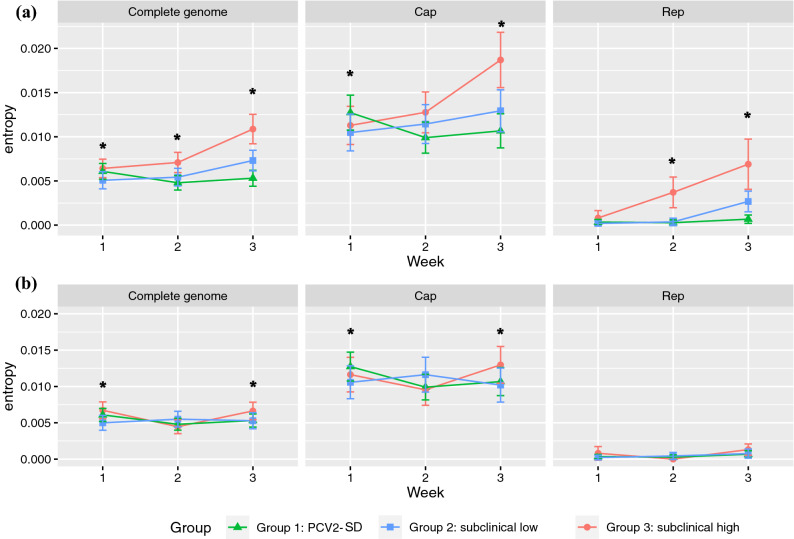
The entropy average and 95% confidence intervals of the complete genome and Cap and Rep coding regions are reported for each wpi. The different groups have been color-coded. The entropy values are reported for the complete dataset **(a)** and after excluding the ‘hypermutant’ subjects (i.e. 153 and 180) **(b)**. Asterisks indicate the presence of significant differences among groups.

The reconstructed viral consensus sequences (based on a majority consensus rule) showed identity with the inoculum sequence, irrespectively of the pig sampled, clinical group and collection time. However, the evaluation of single nucleotide variations (SNV) throughout the whole genome in different groups demonstrated a quite different trend. Despite the within-host viral heterogenicity was similar at 1 wpi, the group with high viral load in serum but no overt clinical signs (Group 3) showed a progressive increase in SNV frequency along the genome over time, with several variable positions shared by different subjects (Fig. 2). On the contrary, an extremely low pathogen genetic variability was detected in diseased pigs (Group 1) over the whole study period, while animals from Group 2 evidenced an intermediate pattern. The results were confirmed by overall entropy evaluation and comparison (Fig. 3a). Since entropy is associated with the amount of disorder in a system, it can represent an effective summary statistic of the overall variability of viral quasispecies within the ‘host system’. A significant difference (*P* < 0.05) between groups at all considered time points was evidenced through Kruskal–Wallis test when analyzing the full genome with, a highly significant difference detected only at 3 wpi (*P* < 0.001). Based on pairwise Mann‐Whitney tests, Group 1 showed a higher entropy compared to Group 2 at 1 wpi (*P* = 0.014), while group 3 had a higher diversity compared to Group 1 (*P* = 0.044) at 2 wpi. At 3 wpi, animals from Group 3 displayed a higher entropy compared to Group 2 (*P* = 0.034) and 1 (*P* < 0.001), while a significantly higher entropy was observed in Group 2 compared to Group 1 (*P* = 0.007). The animal #180 from Group 3 showed the same mutations at different wpi, while most of the SNVs were detected at only one sampling point for the other animals (Supplementary Fig. 1).

The analysis to estimate the variations in entropy over time were also done for ORF1 and ORF2, independently. The Cap coding region (ORF2) showed the higher average entropy for all groups following the same trends over time (Fig. 3). Kruskal–Wallis test evidenced a significant difference between groups at both 1 wpi *(P* = 0.014) and 3 wpi (*P* < 0.001). Specifically, a significant difference was found at 1 wpi between Groups 1 and 2 (*P* = 0.025), and at 3 wpi between Groups 2 and 3 (*P* = 0.026) and Groups 1 and 3 (*P* < 0.001). Although the *Rep* coding region demonstrated an overall lower entropy, the between-group difference became even more evident compared to other regions. Kruskal–Wallis test evidenced a significant difference at 2 and 3 wpi (*P* < 0.001). Group 3 was statistically different form Group 2 (*P* = 0.002) and Group 1 (*P* < 0.001) at 2 wpi. At 3 wpi, Group 1 was significantly different from Group 2 (*P* = 0.001) and Group 3 (*P* < 0.001).

The relationship between antibody titers and entropy was also explored. Despite a certain correlation tendency was observed (Supplementary Fig. 2), the association was not statistically significant.

### Individual level entropy

**Figure 4:**
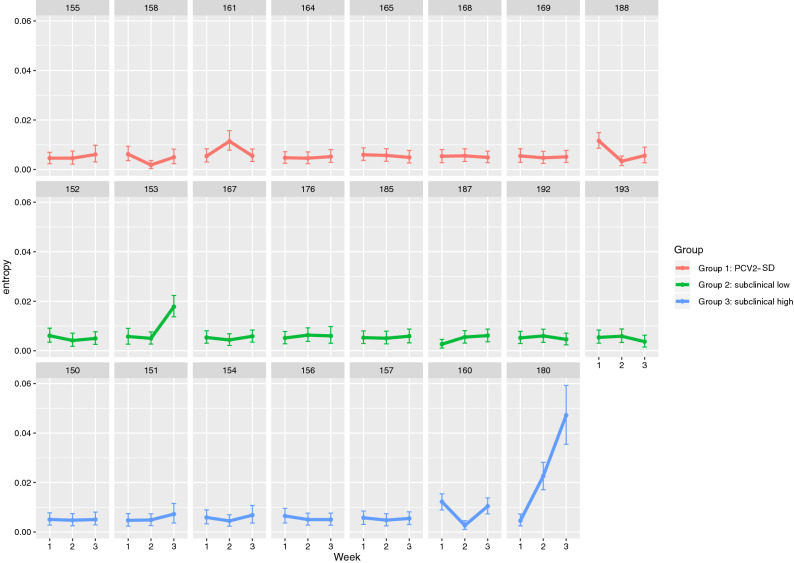
The entropy average and 95% confidence intervals of the complete genome are reported for each subject and wpi. Different groups have been color-coded.

A pig by pig entropy analysis was also performed to explore the within-host viral heterogeneity. A remarkable heterogeneity among animals was observed, especially in animal 153 (from Group 2) which showed a relevant entropy increase at week 3, and animal 180 (from Group 3) which showed this increased variability in viral subpopulations compared to the other subjects starting from 2 wpi. (Fig. 4). A more detailed analysis revealed that the difference was mainly focused on the Rep coding region of these subjects (Supplementary Fig. 3). In fact, when these subjects were excluded, the entropy difference among groups remained true for the Cap region, while the difference became less evident in the Rep gene region, although the Group 3 still demonstrated the higher variability at 3 wpi (Fig. 3b).

## Discussion

The presence of a large within-host viral heterogeneity, often referred as quasispecies, has been demonstrated for several viruses. The continuous generation of new variants can play a relevant role in viral biology, persistence and pathogenesis, allowing viruses to either modify their cell and host tropism or to overcome inhibiting factors like host immune response or antiviral therapies^24,25^.

Currently, the vast majority of available knowledge has been obtained from a limited number of chronic/life-lasting infectious diseases caused by RNA viruses, like the Human Immunodeficiency Virus, Hepatitis C Virus, Hepatitis B Virus^26^. A lower amount of data obtained from acute infections (still essentially caused by rapidly evolving RNA viruses) has demonstrated the existence of a noteworthy intra-host variability and suggested its effect on viral evolution and disease features^19,22,27^. However, the relationship between the mutant spectrum and the clinical outcome is often unclear, poorly understood and hardly generalizable. In fact, both positive and negative correlation between quasispecies diversity and disease severity have been described with contradictory results^19,22^.

Features of viral quasispecies from single-stranded circular DNA viruses like PCVs have been less investigated, especially for animal viruses. Their evolutionary potential should be of higher interest to human health and farm profitability, since the intensive farming determine favorable conditions for the generation of huge and interconnected viral mutant clouds, which allows for a rapid exploration of the fitness landscape and leads to a remarkable adaptive potential to new environments. Significantly, global pork production has increased by more than 80% between 1985 and 2010, which went with a modification in the productive system and the emergence and discovery of tens of pathogens and pathogens variants^28^. A recent study has investigated the presence, features and epidemiological relevance of PCV2 quasispecies in field conditions^16^. Still, the linkage between intra-host heterogeneity and PCVD has never been investigated.

The present study, performed in more controlled experimental settings, allowed to highlight two main results: (1) the presence of a significant difference in quasispecies extent depending on viremia level and clinical signs emergence; (2) the existence of ‘hypermutant’ subjects, harboring a remarkably higher number of viral genetic variants. Animals with no clinical signs demonstrated an increased within-host viral heterogeneity, which was particularly evident in highly viremic subjects. On the contrary, the viral genetic variability of pigs developing overt clinical signs, clearly decreased from 2 wpi until the end of the study, although it was comparable to the other groups at the beginning (1 wpi). These data suggest a likely interaction between viral population diversity and disease severity, as reported for other pathogens^19,22,29^.

The *Cap* gene region was characterized by a higher entropy, posing in favor of a pivotal role of the host immune response in shaping viral heterogeneity^7,15,16^. PCV2 pathogenesis is deeply bound to the host immune system. In fact, while an effective immune response can control PCV2 infection, the virus relies on actively replicating cell for its own replication. The direct consequences of viral replication, especially in immune cells, and the deregulation of immune signaling can cause a severe immunosuppression in infected animals. Actually, PCV2-SD is characterized by a significant lymphocyte depletion in lymphoid tissues and alteration of cytokine and chemokine expression profile^17,18^. Based on these premises, two components are likely to contribute to the observed scenario: the viremia level (i.e. the effective population size) and the immune system effectiveness (i.e. the selective pressure). Because of the high mutation rate, a more pronounced replication could easily justify the marked viral heterogeneity observed only in highly viremic animals. However, the totally different entropy pattern observed in clinical (Group 1) and sub-clinical animals (Groups 2 and 3) suggests that other forces must be in action. We hypothesize that the virus-induced immunosuppression in diseased animals may lead to a lower selection coefficient. The absence of significant differences in Ab titers among clinically and subclinically affected pigs appears to contrast this hypothesis. Nonetheless, it must be kept in mind that IPMA are a quite generic measure of host response (total antibodies), and future studies of immune cell population composition and activation should be performed to give light on this hypothesis. Although it is known that IPMA antibodies correlate positively with neutralizing antibodies (NA), pigs with higher viral loads tend to have lower titers of NA^30^. Therefore, it cannot be ruled out that differences in NA titers would have been detected if measured.

A potential alternative hypothesis is the emergence and predominance of a limited number of (or single) more virulent variants, which was responsible for the overt disease in just a subset of animals. However, no variations were observed at consensus sequence level, proving that none of these mutations effectively reached fixation. Similarly, few SNV appeared to persist over time independently of the considered group. Significant perturbations occurring in a mutant-spectra without any modification in the consensus sequence have already been reported, and evolution can therefore be viewed in terms of a disequilibrium and variations of these spectrum^24^. In fact, a viral quasispecies represents a single selection unit where variants are not selected based on their individual fitness, but on the fitness of the overall mutant distribution whose components share the selectable trait^31,32^. The PCV2 scenario herein observed appears to conform to this principle. Pigs with an effective immune system able to avoid disease emergence, are apparently selecting for a broad quasispecies rather than individual escaping variant. When a mutant spectrum harbors a wide repository of variants, selection of genome subsets in a new environment can be very rapid, which could be more beneficial for long-term viral persistence within host, compared to single escape-mutant selection. While the reproductive advantage of the latter could be promptly limited by new antibody or cell response development or due to cross-reactivity in other antigenic sites, a larger quasispecies cloud further enhance the sequence space exploration potential, and thus the likelihood of transitory emergence of at least partially immune escape variants.

The above-mentioned pattern, although less evident, holds true also excluding from the analysis the ‘hypermutant’ individuals: subclinical-high viremic groups demonstrated a more pronounced variability compared to PCV2-SD affected ones at least at 3 wpi (Fig. 3b). The most notable difference is represented by the *Rep* gene, where after ‘hypermutant’ subjects exclusion, the differences among groups becomes essentially negligible with a much lower entropy compared to the *Cap* gene. The causes behind the higher variability observed within certain subjects remains obscure. Even if a higher sequencing error frequency could be hypothesized, the samples were processed using the same methods and sequenced in the same Ion Torrent run, making this scenario unlikely. Previous studies performed on human pathogens have evidenced the occurrence of remarkable differences in within-host variability between individuals^22,33^. Additionally, fluctuations in the genetic composition were proven to occur in viral population replicating in stable environments, suggesting that this disequilibria may provide a selective advantage to viruses that can be then fully exploited in changing environments^34^, like the ones induced by the host immune system or by replication in different cell or tissues. Interestingly, a higher than expected variability was observed in the ORF1 region in both ‘hypermutants’ pigs, potentially leading to alterations in viral replicative capabilities. Although an obvious impact on viral virulence was not observed, being ‘hyper mutants’ pigs asymptomatic animals, the epidemiological role of these subjects must be carefully considered since they could represent an incubator and amplifier of new variants, which might be then transmitted and selected at population level. The present study confirms the quasispecies nature of PCV2 infection, which appears to be affected mainly by a combination of viral population size and immune selection pressure efficacy. Pigs with overt clinical signs, involving immunosuppression, seem to induce much lower selective pressure than healthy animals, leading to quite homogeneous viral populations. Immune pressure may probably act on the whole mutant spectrum breadth and thus on the viral evolution potential and plasticity, rather than on specific mutations or variants. The detection of ‘hypermutant’ subjects will require further investigations on the causes and frequency of this phenomenon, since the epidemiological relevance cannot be underestimated. The obtained data stresses once more the need for controlling PCV2 population size both at individual and population level, which requires the effective implementation of biosecurity measures and the application (and potentially improvement) of vaccination protocols able to constrain the within host replication potential and the consequent risk of new variants emergence.

## Methods

### Experiment design and sample collection

Twenty-three 6 weeks-old nursery pigs from a larger experimental study (unpublished data) were included in the analyses. Prior to inoculation, animals had healthy condition and very low PCV2 antibody titers (≤ 1:80) measured by immunoperoxidase monolayer assay (IPMA)^35^, which were equivalent to ELISA negative values^36^. In addition, pigs were negative to PCV2 by real-time quantitative PCR (qPCR)^23^. After 1 week of acclimation, all pigs were inoculated intranasally (2 mL, half of the volume was administered in each nostril) and intramuscularly (1 mL on the left neck muscles) with 10^4,5^ TCID_50_/animal of a PCV2b isolate (inoculum). Animals were longitudinally monitored for three weeks, with a daily clinical observation. Serum samples, and both nasal and rectal swabs were collected on a weekly basis. Animals were weighted at the inoculation day and subsequently at the necropsy day; average daily weight gain (ADWG) was calculated. All animals were euthanized at three weeks post-inoculation (wpi) with an overdose of barbiturate.

Animal care and study procedures were conducted under the approval of the Ethical and Animal Welfare Committee of the *Universitat Autonoma of Barcelona* and the Animal Experimentation Commission from the local government (Dpt. de Medi Ambient i Habitatge from the Generalitat de Catalunya; Reference 5796). All experiments were performed in accordance with relevant guidelines and regulations.

### Pathological studies

A complete examination of each animal was performed at necropsy and samples of tonsil, spleen, inguinal superficial lymph node and mesenteric lymph node were collected and fixed by immersion in 10% buffered formalin. These lymphoid tissues were scored for LD and GI following a previously described scoring system^37^. In addition, detection of PCV2 genome by ISH was also performed on the same tissues and scored for the amount of viral DNA (absence, low, moderate or high; 0 to 3 respectively) based on Rosell et al*.* (1999)^37^. A combined mean pathological score (from 0 to 3) including LD, GI and ISH scores was generated per tissue and per animal. Values from 0 to 1.5 were considered compatible with a PCV2 subclinical infection (PCV2-SI) and, therefore, were classified as non-PCV2-SD, while values ≥ 1.5 were considered compatible with PCV2-SD.

### Quantitative PCR and serology

#### DNA extraction/qPCR

DNA was extracted from 200 µL of serum or 300 µL of nasal swab suspensions following the Nucleospin Blood (Macherey–Nagel, GmbH & Co KG, Düren, Germany) manufacturer’s instructions. DNA from rectal swab was extracted from 300 µl of swab suspensions according to the QIAamp DNA Stool Mini Kit (Qiagen GmbH, Germany) recommendations. PCV2 was amplified and quantified using as described by Olvera et al. (2004)^23^. PCV2 load is expressed as log_10_ PCV2 DNA copies per mL of serum or PBS (swab suspension). The sensitivity of this technique is between 3–4 log_10_ copies/mL.

#### PCV2 antibody detection

Antibody titers against PCV2 were measured by IPMA following previously described protocols^38^. IPMA results were expressed in Log_2_ titers.

### DNA extraction and high throughput sequencing

DNA from sera samples from selected animals together with the inoculum were extracted using MiniElute Virus kit (QIAGEN) and full PCV2 genome amplification was done following the protocol described before^39^. Briefly, the optimized 50 µl PCR contained 15 pmol of each primer (PCV490F, 5′-TCCGCGGGCTGGCTGAACTTTTGA-3′ and PCV497R 5′-CCCGCGGAAATTTCTGACAAACGT-3′), 0.3 mM dNTPs, 3 µl extracted DNA and 5 U Superfi high-fidelity DNA polymerase (Invitrogen). The amplicons were purified by the QIAquick PCR purification kit (QIAGEN), eluted in 30 µl Tris buffer and quantified and quality assessed by the Agilent High Sensitivity DNA kit (Agilent Technologies). All DNA samples were individually deep-sequenced using Ion-Torrent platform and analyzed to evaluate the within-subject PCV2 variability over time and its association with different clinical outcomes.

### Bioinformatics analysis

Read quality of obtained FASTQ files was visually inspected using PRINTSEQ^40^. Reads that were shorter than 30 bp in length, with an average quality lower than 20 or had more than one base with a quality lower than 10, were filtered out. Additionally, tag removal and trimming of poor-quality bases, poly-N at 5′ and 3′ tails was performed using the same software. Reads were aligned to the PCV2 reference genome (NC_005148) using Bowtie2^41^. Samtools was used to convert and sort the obtained SAM files^42^. Finally, sample specific coverage and consensus sequence were obtained using QUASR^43^.

### Subpopulation study

The discrimination between true mutation and sequencing errors represent one of the main challenge of NGS-based technologies. To evaluate the presence of viral subpopulations, each position-specific single nucleotide variations (SNV) and its frequency was identified with the highly specific LoFreq method^44^, implementing a Poisson–binomial distribution model and accounting for base-call quality value as well as other sources of uncertainty to identify true variants from sequencing errors. The significance level was set to a *P value* < 0.01.

### Statistical analyses

To compare the within sample variability, Shannon-entropy was calculated for each genome position in each PCV2 sample (i.e. for each selected animal and sampling time) using QuRe implementing a Poisson-binomial distribution model for NGS-error correction^45^. The overall entropy was assumed as summary statistic representative of the overall within-host viral heterogeneity. The presence of a statistical difference in entropy level among the defined groups and/or week post-infection was evaluated using the non-parametric Kruskal–Wallis test, followed by post-hoc tests (Mann–Whitney tests) with Bonferroni correction. A similar approach was used to evaluate the presence of statistical difference in viral loads and antibody titers among defined groups over at different time points. The relationship between viral load and antibodies titers and average entropy was also assessed calculating the Spearman correlation coefficient, for each sampling time and on the whole study duration. The statistical significance level was set to *p* < 0.05.

## Supplementary information

Supplementary Information.

## Data Availability

The entire sequence dataset is available in the Sequence Read Archive (SRA) database with submission number SUB6925781. The consensus sequence used is available at Genbank with accession number MT951192.
