# Supplementary material for: Porcine circovirus 2 (PCV2) population study in experimentally infected pigs developing PCV2-systemic disease or a subclinical infection
Source: Sci Rep. 2020 Oct 20;10:17747. doi: 10.1038/s41598-020-74627-3 (PMC7576782; doi:10.1038/s41598-020-74627-3)
Supplement: Supplementary file 1 — Supplementary Information. [file 41598_2020_74627_MOESM1_ESM.pdf]

Porcine circovirus 2 (PCV2) population study in experimentally infected pigs developing PCV2-systemic disease or a subclinical infection

Florencia Correa-Fiz[a,b,$,*], Giovanni Franzo[c,$], Anna Llorens[a,b], Eva Huerta[a,b], Marina Sibila[a,b], Tuija Kekarainen[a,#], Joaquim Segalés[a,b,d].

[a]Centre de Recerca en Sanitat Animal (CReSA, IRTA-UAB), IRTA, Bellaterra, Spain

[b]OIE Collaborating Centre for the Research and Control of Emerging and Re-emerging Swine Diseases in Europe (IRTA-CReSA), Bellaterra, Barcelona, Spain

[c]Department of Animal Medicine, Production and Health (MAPS), University of Padua, Legnaro (PD), Italy.

[d]Departament de Sanitat i Anatomia Animals, Facultat de Veterinària, UAB, Bellaterra, Spain.

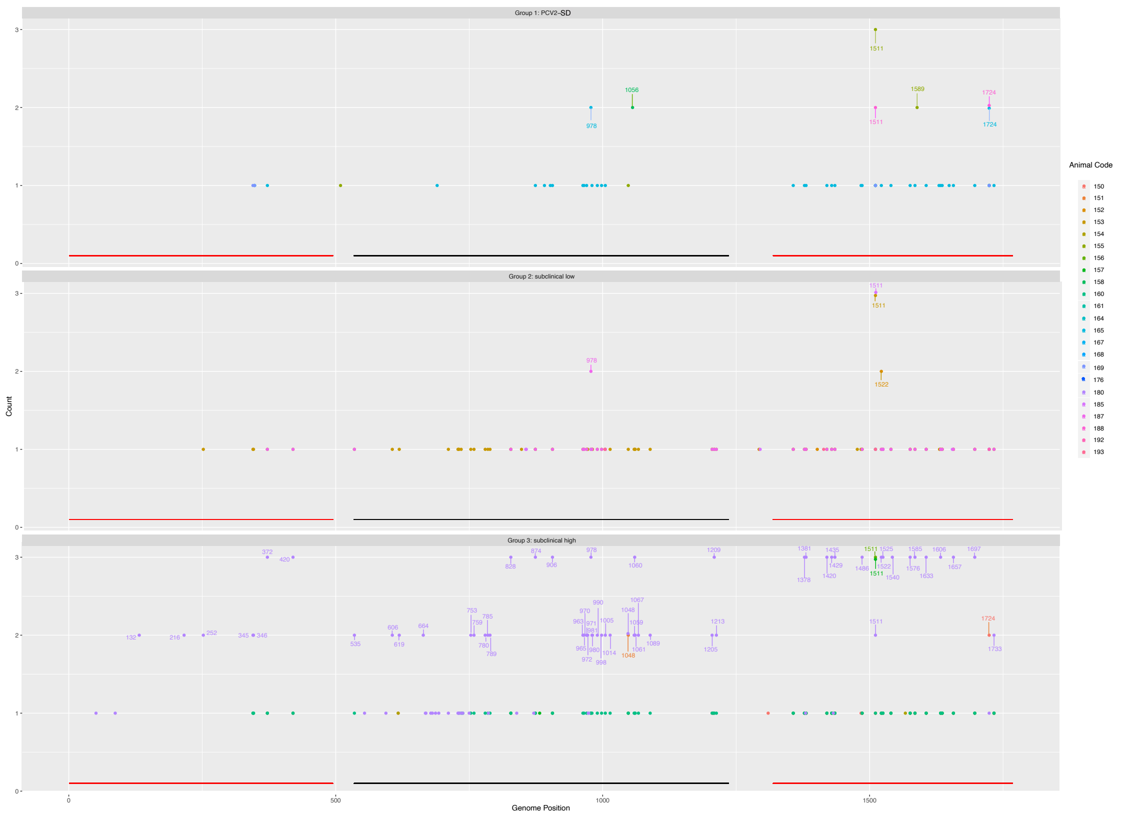

Supplementary figure 1. Scatterplot reporting the count of weeks in which a specific SNV has been detected. When a SNV was identified for more than one week, the respective genome position is reported. Subjects have been depicted with different colors, while the groups are reported in separate rows. Black and red lines at the bottom represent Cap and Rep regions, respectively.

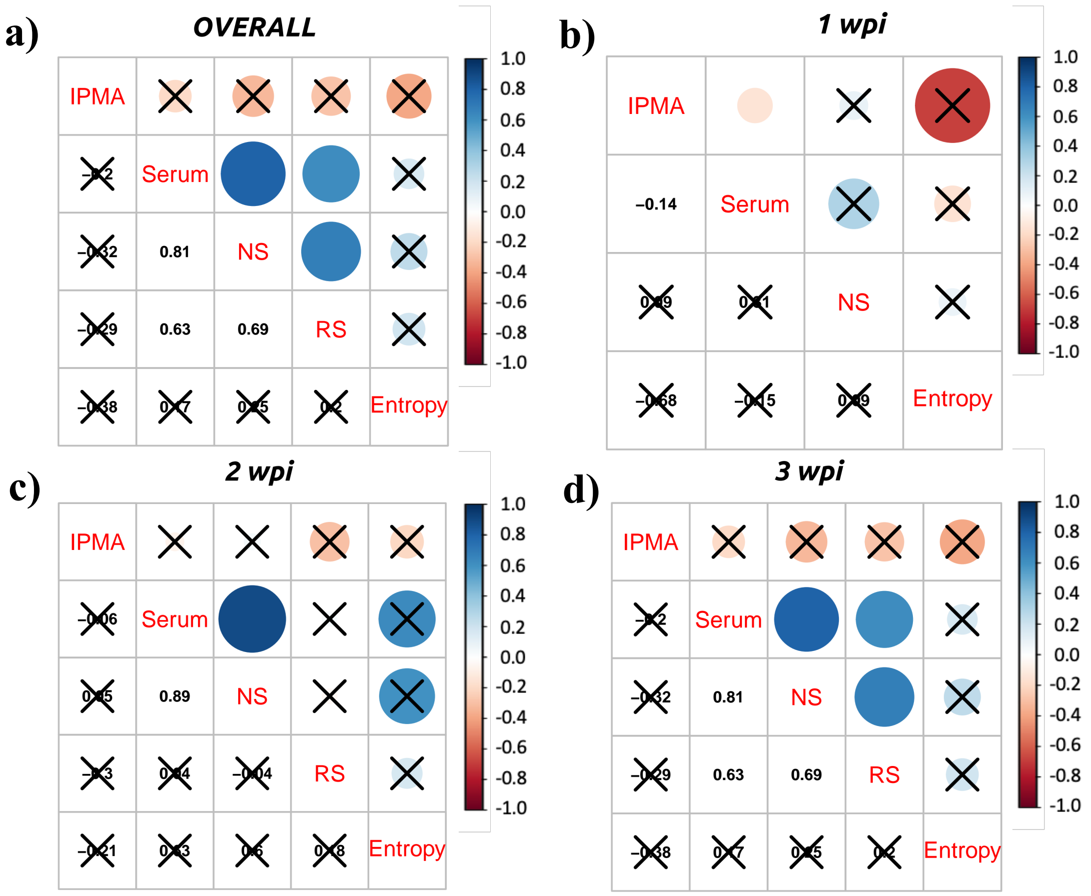

Supplementary figure 2. Correlation plots describing the correlation between pairs of variables analyzed over the whole experiment duration (a) and for one (b), two (c) or three (d) wpi, independently. The Spearman correlation coefficient is reported both numerically (lower triangular portion of the matrix) and graphically (upper triangular portion of the matrix): size and color of the circles is proportional to the coefficient. A cross indicate the absence of statistical significance.

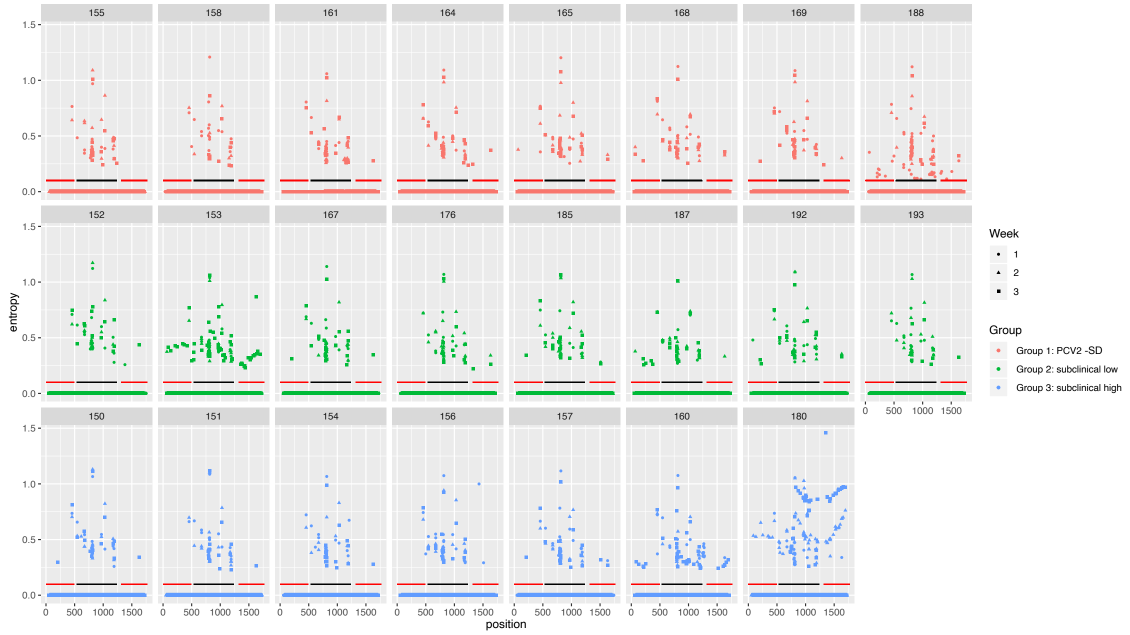

Supplementary figure 3. Scatterplot reporting the entropy values for each PCV2 genome position. Subjects are depicted in independent cells, while wpi are described with different point shapes. Different groups have been color-coded and are shown in different rows. Black and red lines at the bottom represent Cap and Rep regions, respectively.
